# Ictal epileptic headache: an old story with courses and appeals

**DOI:** 10.1007/s10194-012-0485-y

**Published:** 2012-09-27

**Authors:** Pasquale Parisi, Pasquale Striano, Andrea Negro, Paolo Martelletti, Vincenzo Belcastro

**Affiliations:** 1 NESMOS Department, Faculty of Medicine and Psychology, Child Neurology, Headache Paediatric Center, Paediatric Sleep Disorders, Chair of Paediatrics, Sapienza University, c/o Sant’Andrea Hospital, Via di Grottarossa, 1035-1039 Rome, Italy; 2 Pediatric Neurology and Muscular Diseases Unit-DINOGMI-Department of Neurosciences, Rehabilitation, Ophtalmology, Genetics, Maternal and Child Health, University of Genoa, G. Gaslini Institute, Genoa, Italy; 3 Department of Clinical and Molecular Medicine, Faculty of Medicine and Psychology, Regional Referral Headache Centre, Sant’Andrea Hospital, Sapienza University, Rome, Italy; 4 Stroke and Neurovascular Regulation Lab, Department of Radiology, Harvard Medical School, Massachusetts General Hospital, Boston, MA USA; 5 Department of Neuroscience, Neurology Clinic, Sant’Anna Hospital, Como, Italy

**Keywords:** Cortical spreading depression, Headache, Migraine, Epilepsy, Ictal epileptic headache

## Abstract

The term “ictal epileptic headache” has been recently proposed to classify the clinical picture in which headache is the isolated ictal symptom of a seizure. There is emerging evidence from both basic and clinical neurosciences that cortical spreading depression and an epileptic focus may facilitate each other, although with a different degree of efficiency. This review address the long history which lead to the 'migralepsy' concept to the new emerging pathophysiological aspects, and clinical and electroencephalography evidences of ictal epileptic headache. Here, we review and discuss the common physiopathology mechanisms and the historical aspects underlying the link between headache and epilepsy. Either experimental or clinical measures are required to better understand this latter relationship: the development of animal models, molecular studies defining more precise genotype/phenotype correlations as well as multicenter clinical studies with revision of clinical criteria for headache-/epilepsy-related disorders represent the start of future research. Therefore, the definition of ictal epileptic headache should be used to classify the rare events in which headache is the only manifestation of a seizure. Finally, using our recently published criteria, we will be able to clarify if ictal epileptic headache represents an underestimated phenomenon or not.

## Historical background

That “migraine in the borderland of epilepsy” has been recognized since Sir Gowers’ famous book published in 1907 [1]. In an epoch before electroencephalography (EEG), Gowers most likely stated: “…the most frequent relation of migraine to epilepsy is as source of error;….in extremely rare instances one affection may develop while the other goes on”. More than 100 years later, in the era of digital EEG recordings, we are firmly reporting that sometimes “migraine itself can even be epilepsy”: the overlap being partial or complete, not always synchronous (being mainly a peri-ictal phenomenon), but, in certain cases (probably largely underestimated), “the headache represents the only ictal phenomenon”, and recently, we named this condition “ictal epileptic headache” (IEH) [2]. In particular, IEH is recognized as a headache (“as sole ictal epileptic manifestation”) lasting from minutes to days with evidence of ictal epileptiform EEG discharges, which resolves after intravenous antiepileptic medications [2] (Table 1).

**Table 1:** Proposed criteria for ictal epileptic headache (IEH)

Diagnostic criteria A–D should all be fulfilled in order to make the diagnosis “IEH”
A. Headache^a^ (as sole ictal epileptic manifestation) lasting minutes, hours or days
B. Headache, ipsilateral or contralateral to lateralized ictal epileptiform EEG discharges (if EEG discharges are lateralized)
C. Evidence of epileptiform (localized^b^, lateralized or generalized) discharges on scalp EEG synchronous to headache complaints; different types of EEG anomalies can be observed (generalized spike-and-wave or polyspike-and-wave, focal or generalized rhythmic activity or focal subcontinuous spikes or theta activity intermingle or not with sharp waves) with or without photoparoxysmal response (PPRs)
D. Headache resolves immediately (within a few minutes) after i.v. antiepileptic medication

^a^A specific headache pattern is not required (migraine with or without aura, or tension-type headache are all admitted)

^b^Any localization (frontal, temporal, parietal, occipital) is admitted

In this review, the terms headache and migraine are used interchangeably, as in pediatric age it is often impossible clinically to distinguish migraine from other forms of pediatric headache (e.g., tension-type headache). It is also important to stress that IEH has to be included among “secondary headache”, being-by definition-an “ictal epileptic manifestation”. Yet, being a “secondary headache”, it can also have similar but not typical migraine features; moreover, family history of epilepsy and headache, as risks factors, are often associated.

Since the 1950s, there have been described cases from German [3], English [4] and Italian [5, 6] literature, suggesting that “headache” can just be “an epileptic headache” and “…. it can be even the only clinical manifestation of idiopathic epilepsy” [5]. Thus, the concept of ictal headache is indeed old [3–6]. However, in the 1960s the term “migralepsy” was coined [7] which has been permeating the epilepsy and headache culture till now. Migralepsy comes literally from combining the words migraine and epilepsy. This term was introduced to describe a condition wherein a migraine with aura attack is followed by symptoms characteristic of epilepsy. To make a diagnosis of migralepsy, a temporal relationship between the migraine aura and a seizure event (within an hour) is necessary.

With regard to migralepsy cases from literature, recent articles [8–11] have provided a clear demonstration of the inadequacy of the current ICHD criteria definition of migralepsy. After the first “migralepsy” concept by Lennox and Lennox [7], during the 1980s [12, 13] and more recently till now [14, 16–23], an increasing number of “ictal headaches” have been reported. Consequently, we have suggested [2, 10, 11, 17, 23–33] that the “migralepsy sequence” may not exist at all and that the initial part of the “migralepsy sequence” may be simply an “ictal epileptic headache” [2] followed by other ictal autonomic and/or sensory and/or motor and/or psychic features.

## Emerging physiopathological aspects

It has been stressed that hyperexcitation occurs in epilepsy, while in migraine a brief hyperexcitation period (depolarization) is followed by a long hypoexcitation period (spreading depression), followed again by hyperexcitation, as rebound phenomenon [34–36]. Moreover, a *disexcitability* (hyper- and hypoexcitation in the same migrainous patient at different points in time) condition has even been demonstrated [37, 38].

Migraine pathophysiology is still controversial [34–40]. In fact, although cortical spreading depression (CSD) has been shown to activate the trigeminovascular system, whether seizures or CSD causes true migraine typical attack remains a matter of debate. Nevertheless, CSD seems to be the connecting point between migraine and epilepsy [35, 39, 40]. It is characterized by a slowly propagating wave (2–6 mm/min) of sustained strong neuronal depolarization that generates transient intense spike activity as it progresses into the brain tissue (resulting in a transient loss of membrane ionic gradients and in a massive surge of extracellular potassium, neurotransmitters and intracellular calcium), followed by neural suppression which may last for minutes. The depolarization phase is associated with an increase in regional cerebral blood flow, whereas the phase of reduced neural activity is associated with a reduction in blood flow [39].

The trigeminovascular theory [41] is nowadays the most widely accepted theory in the physiopathology of migraine. CSD would be able, as more recently demonstrated [42], to constitute a nociceptive stimulus capable of activating peripheral and central trigeminovascular neurons in the spinal trigeminal nucleus (C1–C2) that underlie the headache pain [42]. In other words, a wave of spreading depression in the visual cortex can induce nociceptive signals in the overlying meninges, resulting in sequential activation of peripheral (first-order) and central (second-order) neurons of the trigeminovascular pathway, which is a likely mechanism of migraine headache.

In particular, the possible correlation between CSD and migraine with aura (MA) [41–44] was first investigated, whereas even in patients suffering from migraine without aura (MoA), the presence of CSD in silent cortical areas [45, 46] as an underlying possible mechanism has been hypothesized. It should be kept in mind that CSD is not a phenomenon that is strictly linked to the cortical structures. Cortical and subcortical areas appear to be hierarchically divided according to how likely they are to develop CSD, though the occipital lobe appears to be the most likely area [23, 24, 28, 45, 47]. Therefore, in the central nervous system, this hierarchical organization based on “neuronal networks” (cortical and subcortical) may be more or less prone to CSD (migraine) and epileptic focal discharges (seizures) [23, 24, 28, 46–48].

How CSD and epileptic discharges can, in more detail, facilitate each other, although with different degree and efficiency? In other words, why could the onset of epileptic seizure facilitate the onset of CSD to a greater degree than the onset of CSD facilitating the onset of epileptic seizure? In this respect, we would like to have a look, deeply, in more detail, at recent experimental and clinical literature data on this topic.

The most interesting data about genetic defects leading to both epilepsy and migraine are regarding familial hemiplegic migraine (FHM) [49, 50]. The FHM1 gene CACNA1A codes for the pore-forming subunit of Ca_v_2.1 P-/Q-type calcium channels [51–53] and its mutations might very well influence CSD, since P-/Q-type calcium channels mediate glutamate release in cortical neurons [52]. The FHM2 gene ATP1A2 [54] codes for the α_2_ subunit of sodium/potassium ATPase, responsible for pumping potassium ions into the cell and sodium ions out of the cell [55]. Mutations have recently been found in FHM families (FHM3), in the SCN1A gene located on 2q24, already known to be associated with epilepsy [56]. SCN1A mutations can also cause genetic epilepsy with febrile seizures plus (GEFS+), severe myoclonus epilepsy of infancy (SMEI) and some other rare epilepsy syndromes [57]. There are insufficient genotype–phenotype correlations in FHM, according to the different possible mutations. For example, FHM1 mutations were also found in family members with migraine only. This suggests that gene mutations for FHM may also be responsible for the common forms of migraine, probably due to different genetic and non-genetic modulating factors [58].

With regard to the “cortex *disexcitability*” in migraine subjects [37, 38], new advances now support this point of view [59]. In fact, considering the specific polysynaptic inhibitory sub-circuit involving fast-spiking (FS) interneurons and pyramidal cells (PC) that have been investigated in the FHM1 mice [59], the gain of function of glutamate release at the recurrent synapses between pyramidal cells would certainly increase network excitation; in contrast, the gain of function of glutamate release at the PC–FS synapses would lead to enhanced recruitment of interneurons and enhanced inhibition. This analysis, even though restricted to a specific sub-circuit, makes the important point that the differential effect of FHM1 mutations on excitatory and inhibitory neurotransmission may produce overexcitation in certain brain conditions, but may leave the excitation–inhibition balance within physiological limits in others, thus explaining the episodic nature of the disease with alternate hyperexcitation and hypoexcitation in the same subject at different time (supporting thus the *disexcitability* concept in migraine subjects).

A plausible hypothesis explaining the clearly different degree and efficiency for activating each other is that the initiation mechanisms of CSD and seizure are similar, but the evolution is different depending on whether the neuronal hyperactivity and consequent increase in (K+) exceed a critical level that causes self-regeneration of the depolarization; in this hypothesis, CSD represents ‘‘a poorly controlled seizure’’ in which (K+) regulation is completely disrupted [59, 60]. Indeed, in this regard, local neuronal hyperactivity progressively recruiting a synchronous discharge via recurrent excitatory collaterals and (K+) accumulation has been proposed to initiate epileptic discharge in slice models [61]. CSD, experimentally induced in rats, increases cortico-cortical evoked responses and strongly induces “brain-derived neurotrophic factor” with synaptic potentiation in vivo [62] and the induction of a “long-term potentiation-like” (LTP-like) phenomenon by CSD receives support from experimental evidence. Also, there are also in vivo data reinforcing the idea of a CSD-induced LTP-like phenomenon [63]. Another recent and intriguing finding about CSD propagation is the model based on interstitial (K+) diffusion, initiating in adjacent dendrites the positive feedback cycle that ignites CSD, in contrast to the hypothesis that CSD propagates through gap junctions. In particular, the opening of the gap junctions would not be required for CSD propagation, but is rather necessary for extracellular homeostasis after CSD [64].

Using an in vitro model of CSD [59], a causative link between enhanced glutamate release and CSD facilitation has been shown. The synapse-specific effect of FHM1 mutations points to disruption of excitation–inhibition balance and neuronal hyperactivity as the bases for episodic vulnerability to CSD ignition in migraine. This finding provides direct evidence that the gain of function of glutamate release at synapses onto pyramidal cells may explain the facilitation of experimental CSD in FHM1 mutant mice, and thus provides novel insights into the controversial mechanisms of CSD initiation and propagation. These data are consistent with and support a model of CSD initiation, in which activation of pre-synaptic voltage-gated Ca+ channels with consequent release of glutamate from recurrent cortical pyramidal cell synapses and activation of NMDA receptors are key components of the positive feedback cycle that ignites CSD. Moreover, the role in particular of different voltage-gated Ca^2+^ channels in CSD has recently been investigated [65]. After blockade of either the P-/Q-type Ca^2+^ channels or the NMDA receptors, CSD cannot be induced in wild-type mouse cortical slices. In contrast, blockade of N- or R-type Ca^2+^ channels has only a small inhibitory effect on CSD threshold and velocity of propagation. These findings support a model in which Ca^2+^ influx through pre-synaptic P-/Q-type Ca^2+^ channels with consequent release of glutamate from recurrent cortical pyramidal cell synapses and activation of NMDA receptors are required for initiation and propagation of the CSD involved in migraine [59, 65].

Temporal and spatial associations of CSD and seizures using electrocorticographic (ECoG) recordings in patients with acutely injured cerebral cortex have been examined [35]. The authors reported clinically overt seizures only in one patient and each patient with CSD and seizures displayed one of the following four different patterns of interaction between CSD and seizures: (a) in four patients, *CSD was immediately preceded by prolonged seizure* activity; (b) in three patients, the two phenomena were separated in time and *multiple CSDs were replaced by ictal activity*; (c) in one patient, *seizures appeared to trigger repeated CSDs* at the adjacent electrode; (d) in two patients, *ongoing repeated seizures were interrupted each time CSD occurred.* The reported four patterns were consistent within recordings from the same patient, but differed between patients.

Of particular interest are patients 3 and 4 reported by Fabricius et al. [35] whose *seizure activity spread from electrode to electrode at the same slow speed as CSD,* but preceded it by several minutes. This is noteworthy, since the seizure activity under other conditions spreads much faster than a CSD. To better understand the relevance of this latter finding, it should be stressed that…. “A race car as “Ferrari” can run at a speed of a ‘Fiat 500’ but not vice versa”. This point of view could explain why the onset of epileptic seizure facilitates the onset of CSD to a greater degree than the onset of CSD facilitating the onset of epileptic seizure. The first (Ferrari) usually prefers to use the highways (myelinic) and the latter (Fiat 500) mainly uses the roads (amyelinic), although it is important to stress that a “Ferrari” can easily follow the roads (amyelinic) usually covered by a “500 Fiat”, while the reverse is not true. Accordingly with the above reflections, it is of note that *the patterns recorded by* Fabricius et al. [35] *were consistent within recordings from the same patient, but differed between patients*: highways (myelinic) and little roads (amyelinic) in the same patient usually do not change so much, at least during a not too long period of time.

Yet, another important finding from Fabricius et al. [35] which confirmed our point of view [2, 16, 23–29] is that, in their sample, CSD was more often encountered than seizures, since there were twice as many patients with CSD/peri-infarct depolarization alone than with CSD/peri-infarct depolarization plus seizures. Also, 10 of 11 patients with seizure activity also had CSD, and clinical overt seizures were only observed in 1 of the 11 patients, while seizures were not suspected on clinical grounds in the other 10 patients.

Interestingly, in the described so-called IEH [12–23] case reports, patients are, both, idiopathic (photosensitive or not) and symptomatic; often, they also present a clinical history (personal and/or familial) of epilepsy and migraine. In the cases of positive photo-paroxysmal response, the intermittent photic stimulation evokes headache and they can also have visually induced seizures (Table 1) [2]. With regard to the EEG abnormalities recorded in “ictal epileptic headache” cases [2, 12–23], the same wide spectrum of different EEG patterns (spike-wave activity, ‘‘theta’’ or even ‘‘delta’’ shape, without any spike activity) associated with both CSD and/or seizures were also confirmed “in vivo” by electrocorticography [35].

## Drawbacks: the current ictal epileptic headache definition will inevitably underestimate the phenomenon

We have been suggesting that headache be classified as an isolated ictal epileptic manifestation since 2007 [2, 10, 11, 16–18, 23–33]. The proposed criteria are reported in Table 1. Nonetheless, we would also like to stress that the IEH criteria inevitably underestimates this ictal “autonomic” phenomenon. Thus, besides highlighting the strengths of “our forthcoming criteria”, we would also like to point out “their inevitable drawbacks”.

To date, headache and epilepsy classifications have ignored each other [66]. In the ILAE classification, headache is considered exclusively as a possible semiological ictal phenomenon among the “*non*-*motor*” (point 2.0) features. In particular, headache is described as a “*cephalic*” sensation (sub-classified at sub-point 2.2.1.7) and is not considered as the sole ictal expression of an epileptic seizure. Moreover, headache is not classified as a “pain” (among the “somatosensory” features at 2.2.1.1) or “autonomic” sensation (2.2.1.8), whereas signs of involvement of the autonomic nervous system, including cardiovascular, gastrointestinal, “*vasomotor*” and thermoregulatory functions, are classified as “autonomic” features. Now, although still considered a controversial issue, we must consider that headache pain could in fact originate in the terminal nervous fibers (“*vasomotor*”) in cerebral blood vessels; consequently, headache should be classified as an “autonomic” sensation in the ILAE Glossary and Terminology. Headache could thus be interpreted as the sole expression of an epileptic seizure and classified as an autonomic seizure. To explain why headache may be the sole ictal epileptic symptom, we previously suggested **[** 2, 10, 11, 16–18, 23–33] that an autonomic seizure (i.e., in IEH cases) remains purely autonomic if ictal neuronal activation of non-autonomic cortical areas fails to reach the symptomatogenic threshold, as previously described for other ictal autonomic manifestations in Panayiotopoulos syndrome [67].

In addition, we believe that the social stigma attached to epilepsy may explain a general reluctance (25) (not only in the general public, but even among physicians) to recognize the growing number of documented cases of IEH [2, 10, 11, 16–18, 23–33].

Another notable point is that while unequivocal epileptiform abnormalities usually point to a diagnosis of epilepsy, the lack of clear epileptic spike-and-wave activity is frequent in other ictal autonomic manifestations, as well as in patients with a deep epileptic focus arising, for example, from the orbitomesial frontal zone [68]. In such cases, ictal epileptic EEG activity may be recorded from the scalp or exclusively by means of deep stereo-EEG recording.

An additional point deserving attention is the lack of a clear, repetitive EEG headache-associated pattern, since the ictal EEG recording in such patients does not yield a specific EEG picture. Indeed, different patterns have been recorded during migraine-like complaints in both symptomatic and idiopathic cases [10, 11, 28]. Moreover, when EEG anomalies are recorded, no specific cortical correlations emerge (e.g., focal frontal, parietal, temporal, occipital and primary or secondary generalized) [10, 11, 28].

Lastly, the criteria we propose do not offer the possibility of confirming all suspected cases of IEH by means of intravenous anticonvulsant administration, just as it is not always possible for other types of epileptic seizures; in fact, although in case of “autonomic seizures” such as in IEH, the clinical response seems to be present in almost all published cases, we cannot be sure that i.v. anticonvulsant administration is able to stop a seizure in any cases in these types of patients.

For all the aforementioned reasons, we firmly believe that the diagnosis of IEH (even according to our proposed new criteria) will remain an underestimated phenomenon owing, in particular, to:

- the psychosocial stigma attached to this disease;
- the fact that IEH cannot always be detected from the scalp;
- IEH could rarely be responsive to antiepileptic i.v. administration, as can happen for other type of seizures.

## Conclusion

The clinical pictures of IEH seem to be extremely rare [2] and it has been documented in about 12 cases [12–14, 16–23]. Since its epileptic nature can be documented only with ictal EEG recording and simultaneous intravenous antiepileptic administrations, it is difficult to obtain firm conclusions about the frequency of IEH on epidemiological studies. In this regard, we have recently published an “editorial” completely dedicated to these epidemiological aspects, their possible biases and the underestimation potentially related particularly to pediatric age [69]. Based on the current knowledge and clinical experiences reported, migralepsy (coded in ICHD-II as 1.5.5 “migraine-triggered seizure”) is highly unlikely to exist as such. We therefore propose to take from the Appendix of International Headache Disorders Classifications this term until clear evidence is provided of its existence.

“Ictal epileptic headache” criteria [2, 28, 69] (Table 1) should be used to classify the rare events in which headache can represent the sole ictal epileptic manifestation.

“These findings further highlight the important role of EEG recording in patients with headache, which has been traditionally opposed by the ancestral fierce adversity (25) against the possible link between headache and epilepsy”. Rather, we certainly should think deeply about the inappropriate and exaggerated overuse of the brain CT in the pediatric emergency room in children admitted simply for idiopathic or more frequently “upper respiratory infections”-associated headache.

In conclusion, using our proposed new criteria (Table 1) [2] in a large pediatric population, we will be able to clarify if “ictal epileptic headache” is really a phenomenon that shows a marginal role or, vice versa, represents an underestimated event [68–70].
